# Ethical Challenges in Scientific Studies Within One Health

**DOI:** 10.1007/s40572-026-00522-0

**Published:** 2026-01-26

**Authors:** Henrik Lerner

**Affiliations:** Department of health care sciences, Marie Cederschiöld University, P.O. Box 11189, SE-100 61 Stockholm, Sweden

**Keywords:** Ethics, One health, Planetary health, EcoHealth, One welfare, Health

## Abstract

**Purpose of review:**

The purpose of this short literature review is to present the state of art in One Health ethics, a new field working with ethics in One Health approaches. These approaches have focused on promoting health for humans, animals, plants, and ecosystems mainly in a scientific way. Ethics has often been left out. The goal is to summarize the main findings of the limited ethics discussion in the field and propose the future way forward for the field.

**Recent findings:**

There have been several calls for ethics, and the main discussion has mainly focused on (1) the ethical imperative in One Health, (2) the ethical value of ecosystems, (3) normative aspects of health, (4) core ethical concepts, and (5) ethical decision models.

**Summary:**

For the next decade this field needs to be fully developed and included as a core science within the One Health approaches. To be able to solve the complex problems these approaches are facing, such as the triple crisis (climate change, pollution, and biodiversity loss), more scholars need to work with One Health ethics, which still is a rather underdeveloped field of ethics. Three future trends for the field of One Health ethics were proposed; 1) to find ethical decision models, 2) to bridge the gap between anthropocentrism, zoocentrism, biocentrism, and ecocentrism, and 3) how to balance valuation between different species, organisms or levels, or ethics and economy.

## Introduction

One Health approaches aim to promote health for humans, animals, plants and ecosystems at the same time. They have been widely discussed since 2004, when the Manhattan Principles [1] were introduced – seen as a starting point for the modern One Health approaches. The 12 Manhattan Principles established the linkage between humans, animals, and ecosystems. One Health approaches are inter- or transdisciplinary with human medicine, veterinary medicine and biology, i.e. mainly natural sciences as core sciences [2]. Humanities are sometimes included, and since 2009 there has been a call for ethics [3]. Still, ethics is not a core science within the approaches [4] and there have been many calls for ethics published ([4]– [5]).

This short literature review will start with analyzing the assumption that ethics is needed. The next step is to present some of the main ethical themes already discussed in the field. Thereafter, I will present some of the upcoming ethical issues. Finally, in my conclusion I propose some possible future trends in ethics in One Health approaches.

## Ethics is Needed

### State of the Art

As a response to the problem that human health issues weren’t solved by public health, we have in the 20th century seen a strong urge to broaden the scope of health. This has most profoundly been manifested in what is called One Health approaches [6]. Sometimes authors claim, as I did in the beginning of this review, that the initial point was the establishment of the Manhattan Principles, when in fact the process was ongoing in several parallel approaches. Drivers were aspects of inclusiveness, holism, and turning down barriers between scientific disciplines or stakeholders. The approaches are therefore aiming for inter- or transdisciplinarity, health for more than humans (animals, plants, and ecosystems), and involving more groups than researchers in the research, such as policy makers, research participants or other stakeholders. Their ideal of problem solving is to include as many disciplines as needed to cover all the aspects of a health problem (not just curing diseases in humans). Problem solving in this case is more than eradicating disease. The solutions to one of the biggest threats to health for all, the triple crisis (climate change, pollution, and biodiversity loss), might imply changing behavior, livelihoods, and global economic flows.

The four main One Health approaches are One Health, EcoHealth, Planetary Health, and One Welfare [2]. There are several more, such as GeoHealth, Conservation Medicine, Zoobiquity, and One Biology, just to mention a few. Why they have different names depends on how the approach values the contribution of different sciences (and if they are included as a core science or a help-science), the different entities (such as humans, animals, plants, and ecosystems), and which kind of health aimed for.

So far, these approaches have been mainly science driven with a focus on human medicine (including nursing, public health, etc.), veterinary medicine, and biology (mainly microbiology, ecology, and environmental science). Among the humanities, social science has entered many approaches and are today more or less core. The EcoHealth approach has also included for example anthropology and ethnobiology.

Ethics has mainly been absent, a very striking observation because two of the core sciences, human medicine and veterinary medicine, have their own fields of practical ethics. There have been several calls for ethics within the approaches, but ethics has not been established as a core discipline. To leave the pristine field, I will now turn to the evolution of ethics within the approaches.

### Calls for Ethics

One of the first call for ethics in One Health approaches came in 2009 [3]. That was a call for equity, but rather analyzing economic and social aspects, than ethical. In the 2010 s an initial discussion of what such an ethics could harbor occurred.

Lerner [7] analyzed the foundation of One Health and found that three strands might be important, (1) the normative study of how included disciplines in inter- or transdisciplinary approaches are valued, (2) the normative aspects of the core concept of health, and (3) the ethical issues linked to the relation of as well as the goal of care.

Verweij and Bovenkerk [8] broadened the discussion by suggesting four areas of concern for an ethic of One Health approaches. These areas of concern were (1) scientific, (2) metaphysical, (3) conceptual, and (4) moral. Lerner’s strands could be included in this framework quite easily. The normative aspects of included disciplines fall into the scientific area of concern. Normative aspects of the concept of health (and other concepts such as stated in Lerner & Berg [2]) will partly fall into the conceptual (how concepts are defined) and partly in the metaphysical (how these normative aspects could be attributed to different entities such as animals, plants, or ecosystems). The ethical issues will fall into the moral area of concern. Therefore, Verweij and Bovenkerk’s framework is the best and most including framework for One Health ethics at the moment.

As a summary of this section the first ten years focused on three different areas, (1) ethical frameworks, (2) values, and (3) ethical issues (see [4] for further references).

## Main Ethical Themes in the Debate

I will now turn to the ethical challenges that have been studied in One Health ethics. These have been (1) the ethical imperative in One Health, (2) the ethical value of ecosystems, (3) normative aspects of health, (4) core ethical concepts, and (5) ethical decision models.

### The Ethical Imperative in One Health

The main road of finding an ethic of One Health approaches has been to empirically analyze how the different One Health approaches are defined and constituted and draw ethical conclusions from this ([4]– [5, 7–17]). Therefore, much of the work falls in the field of empirical ethics.

One of the first questions to ask is, is there a need for a specific One Health ethical framework or could already established ethical frameworks in other ethical fields work? Johnson and Degeling [10] found that both ways will be possible to go, without settling the question. That analysis will therefore be continued here.

In the first way, stick to existing frameworks, there have been suggestions from the field to just expand or adopt existing ethical frameworks to One Health issues [18], for example through a lens of the already established Global Health [15]. I can see that the holistic One Health approaches, with a focus on humans, animals, plants, and ecosystems, might imply that ethical knowledge could be gathered from several subdisciplines (Table 1.).

**Table 1:** Different ethical areas important for one health approaches

Ethical areas
Public health ethics
Medical ethics
Care ethics
Research ethics
Bioethics
Veterinary ethics
Animal ethics
Environmental ethics

**Table:** Box 1. Definitions

Term	Definition
Anthropocentrism	Traditional ethics focusing on humans. Occur in two versions: Strong – only humans have moral value Weak – individuals of other species might have moral value but less than humans
Zoocentrism	Animal ethics. Humans and animals have moral value.
Biocentrism	Environmental ethics. Moral value is placed in all living organisms, including humans, animals, and plants.
Ecocentrism	Environmental ethics. Moral value is placed in species and ecosystems

Others claim that there might be fundamental differences. For example, whereas Global Health seems to be based on intergenerational and global health justice, One Health has its ethical foundation on interdependence, which is something else [14].

Another way is to change the ethical foundation from anthropocentrism towards zoocentrism, biocentrism, or ecocentrism (Box 1). Lederman [9] analyzed definitions of One Health and found that inherent in important definitions of the field is an ethical imperative. One Health promotes the health of humans, animals and ecosystems, and therefore also needs to give these entities value and include them in the ethical analysis. This was later echoed by others [13, 16, 17].

If one follows the ethical imperative inherent in One Health, a biocentric foundation seems to be a more promising start and then one can add specific aspects that stem from One Health. Such attempts have often focused on life itself as the reason for moral standing [13, 16].

Van Patter et al. [12] argue that One Health needs to include feminist, posthuman, and anti-colonial viewpoints in order to avoid being an ethic only for white, privileged people. Western science in One Health might still replicate colonial harms when telling people from other cultures what to do. Intersectionality is an important view to include within One Health approaches [19], and indigenous knowledge, values, and ethics should not be excluded ([20]– [21]).

Finally, considering this discussion above, one could ask if only one theory will fit the ethical imperative. Thompson and List [22] argued for plurality in ethics, in their attempt to coin the term “One Bioethics” for this area of ethics. To reach all aspects of One Health ethics, one might need to construct an ethical framework that bridges the gaps between anthropocentrism, zoocentrism, biocentrism, and ecocentrism [17]. Either one could find a theory that encompasses all -isms (which probably is an impossible challenge if one holds a strict view on meta-ethics) or one can try to find theories more pragmatically from different -isms that might work together in different areas, creating a mosaic of ethics. The latter way has also been suggested by Hinchliffe [23] for the treatment of health definitions in One Health approaches and seems for ethics to be a more promising way [17].

### The Ethical Value of Ecosystems

One core issue has been how to value the environment, especially in terms of ecosystems. In One Health, there are two versions, one narrow with a focus on human and veterinary medicine excluding ecosystem health, and one wide including biological sciences and ecosystem health [2]. There is also a difference between EcoHealth and Planetary Health on how to look at biodiversity. Ecohealth focuses on the three-pillar model (ecological, economic and social sustainability) whereas Planetary health focuses on the anthropocentric Brundtland model [2]. The conflict could be brought down to how to balance health and biodiversity in an ethical analysis [24]. One such conflict could be how to value parasites. Often, parasites have a negative value because they cause harm to the host. If one considers parasites from an ecosystem level, they contribute to a rich biodiversity as well as ecological processes within the ecosystem. In this case, parasites could be positive valued. One must analyze if they always are bad or sometimes good [25].

### Normative Aspects of Health

The normative aspects of the concept of health have been discussed. In One Health approaches, the concept of health could be ascribed to different entities and levels and is described in the health matrix [26]. The entities are humans, animals, plants, and ecosystems, and the levels are individual, population, and ecosystem. Humans and animals are the two entities that are easiest to include. In EcoHealth emphasis is often on ecosystems as entities, as well as population level and ecosystem level in health. In One Health emphasis is often on humans and animals as entities and individual health (even more emphasized in One Welfare [27]).

This difference resembles parts of the conflict between animal and environmental ethics. Animal ethics ascribe value to individuals of human and animals, while environmental ethics ascribe value to systems, such as ecosystems, and groups, such as species. This is one of the toughest divides to bridge in ethics. This indicates that claims of merging these approaches into one might be tough due to fundamental value differences [2].

How normative should one be in the discussion of which definition of health to choose? One could follow a path trying to find a similar definition for all entities [28] or one can argue for a pluralistic view on health [23]. There are several categories of definitions of health that could be suitable for One Health [29]. Lerner [30] made an initial analysis to evaluate which of the definitions of health as balance that could be fruitful. This was further developed by Selter and Salloch [31]. Their conclusion was that finding one theoretical concept would be a very hard task to do, due to the wide range both in species and levels. This also depends on whether one is aiming for a definition that originates from finding a common denominator (bottom-up definitions) or that extrapolates from human conditions (top-down definition) ([29]– [30]). Both ways have problems. In the first case, one could choose some biological traits that are shared by all species with the risk of not grasping the whole picture of health in for example birds, non-human mammals, and humans. In the second case, by diluting the important aspects of human health to cover more species, one might still have a lot of species where health couldn’t be applied. Future research on the concept of health should be performed because the concept is the core value in the One Health approaches.

### Core Ethical Concepts

In the limited discussion on ethical concepts crucial to One Health ethics at least three concepts have gained recognition, equity, resilience, and risk.

One of the earliest calls [3] focused on equity. Two other closely related concepts, justice and fairness, have recently been put forefront ([11]– [12, 19, 32–35]). This has been most elaborated in Gislason and Stephen [34] where they elaborate that equity is a core concept to tackle the global challenges humans, animals, and the ecosystems face. They aim for a viewpoint on health that is interspecies and intergenerational and distinguishes between social, environmental, and ecological justice. In their proposed framework they aim to assess equity on two scales, time (present and future) and equity dimensions (how outcomes will influence, how determinants of health resources are available, and how it is possible to gain from determinants of health resources).

Justice seems to be a core ethical aspect to consider. As mentioned above, justice is further divided into several kinds of justice. The emphasis on different kinds of justice might differ in different research areas and also between ethical areas (Box 1). In public health ethics, environmental justice seems to be important [36]. In research on climate change, models with several kinds of justice seem to be developed which might differ in richness of kinds compared to the traditional view in bioethics (for example ([37], – [38])). Fruitful in the future would be to interdisciplinary analyze these attempts to differentiate justice.

In a consensus view from all the presenters from a research track in the 28th Annual ISDRS Conference *Sustainable Development and Courage: Culture*,* Art and Human Rights* analyzing One Health, Sustainable Development Goals, and ethical conflict, two concepts were found to be influencing the whole discussion [4]. These were risk and resilience. Risk could be defined in several ways but could broadly be a combination of hazard and exposure. Although risk could be a very mathematical concept, the normative aspects of risk were stressed. Ethical valuation influences how one perceives risk as well as how one tries to avoid risks. Resilience, which also has several definitions, need to be a broad concept where we need to normatively help people, animals, and ecosystems being resilient.

### Ethical Decision Models

In the consensus view on ethics in One Health approaches presented in Lerner et al. [4], there was a call for ethical decision models. In more established ethics such as bioethics and public health ethics, one opts for decision models, but they are hard to develop. In One Health ethics, it is much more troublesome, due to the ethical imperative [9]. The ethical imperative argues for an ethic that could combine anthropocentric, zoocentric, biocentric, and ecocentric ethics.

One promising starting point would be dialogue ethics [4, 11, 19] that could be further developed into an ethical decision model. One of the attempts from Planetary Health [11] focuses mainly on information sharing the knowledge holder to the inflicted person when one aims for a change in a community. This view of dialogue might risk ending in a one-sided approach not suitable as a dialogue [17, 39]. Laing et al. [19] aims for a reciprocal dialogue which also includes vulnerable groups.

Lerner [39] develops Vilhjalmur Árnason’s work on dialogue ethics to also be applicable to One Health approaches. He finds four aspects that will facilitate a fair dialogue, namely (1) the pre-framing aspect considering how one will frame the ethical issue, (2) the arena aspect considering where the dialogue will take place, (3) the mandate aspect considering which mandate for decision the dialogue has, and (4) the time aspect considering how much time is offered for the dialogue recognizing cultural differences. These aspects are important to visualize differences that might be present to help one holding for example a feminist, posthuman, and anti-colonial position. A recent suggestion is a two-stage dialogue where a first dialogue (a pre-framing dialogue) sets these four aspects before the real dialogue occurs. In this way the first dialogue can consider such aspects as how to include various perspectives and groups, including vulnerable groups and advocates for plants, animals and ecosystems (see [17] for a full description of this ethical decision model).

## Conclusion – Possible Future Trends

This short literature review has gathered those ethical themes discussed in this rather new field. The ethical inquiry needs to be continued. I can as a result of this short literature review see the following future trends for One Health ethics:

- One needs to find ethical decision models.
- One has to solve how to bridge the gap between anthropocentrism, zoocentrism, biocentrism, and ecocentrism.
- There need to be a focus on valuation, i.e. how to balance, for example.
  - ethical aspects of different species (either as a species or as an individual of a species).
  - health either between different organisms or different levels.
  - ethics and economy.

For the next decade this area needs to be fully developed and included as a core science within the One Health approaches. To be able to solve the complex problems these approaches are facing, such as the triple crisis (climate change, pollution, and biodiversity loss), more scholars need to work with One Health ethics, which still is a rather underdeveloped field of ethics.

## Key references

Papers of particular interest, published recently, have been highlighted as:

* Of importance

** Of major importance

** Lerner H, Berg C. A comparison of three holistic approaches to health: One Health, EcoHealth, and Planetary Health. Frontiers in Veterinary Science 2017: 4:163. doi: 10.3389/fvets.2017.00163.
  - ◦This paper analyzes differences between narrow and wide One Health, EcoHealth, and narrow and wide Planetary Health focusing on scientific disciplines, concept of health and core values. This is a good starting point for references to key theoretical papers in the different approaches.
* Lerner H, Nordquist RE, Lederman Z, Keyel J, Nickel PM, Berg C. Ethics, One Health approaches, and SDGs: conference lessons for an emerging field. Front. Public Health 2024:12:1448409. Doi: 10.3389/fpubh.2024.1448409.
  - ◦This paper stems from a conference where several scholars contributed to the state of art of One Health Ethics. The paper condensates the talks together with a consensus discussion from all speakers on the insights gathered.
* Lerner H. The philosophical roots of the ”One Medicine” movement: An analysis of some relevant ideas by Rudolf Virchow and Calvin Schwabe with their modern implications. Studia Philosophica Estonica 2013:6(2):97–109.
  - ◦One of the first attempts to find an ethical foundation for One Medicine, which is one of the predecessors to the One Health approaches. Insights from this paper could still be helpful in finding a comprehensive framework for One Health ethics.
** Verweij M, Bovenkerk B. Ethical promises and pitfalls of one health. Public Health Ethics 2016:9(1):1–4.
  - ◦ One of the first papers addressing what the field of One Health ethics could be. A valuable contribution and still the most comprehensive one.
* Foster A, Cole J, Petrikova I, Farlow A, Frumkin H. Planetary health ethics. In: Myers S, Frumkin H, editors. Planetary Health: Protecting Nature to Protect Ourselves. Island Press: Washington and Covelo, 453–473, 2020.
  - ◦At the moment, one of the most developed attempts to create an ethic for a One Health approach (Planetary Health).
* Van Patter LE., Linares-Roake J, Breen AV. What does one health want? Feminist, posthuman, and anticolonial possibilities. One Health Outlook 2023:5:4
  - ◦Key paper questioning the traditional white-male western perspective within One Health approaches. Introduces and establishes the framework of feminism, posthumanism, and anticolonialism in this field.
** Lerner H. Ethics for One Health Approaches: A Roadmap for Future Directions. Advancing Global Bioethics 22. Springer, 2025.
  - ◦Analyzes suggestions for ethics and proposes ethical decision models for One Health approaches.
* Laing G, Duffy E, Anderson N, Antoine-Moussiaux N, Aragrande M, Beber CL, et al. Advancing one health: updated core competencies. CABI One Health 10.1079/cabionehealth.2023.0002, 2023.
  - ◦This paper analyzes what core competencies those working with One Health approaches need to embrace. There are also suggestions on ethical matters.
* Selter F, Salloch S. Whose health and which health? Two theoretical flaws in the One Health paradigm. Bioethics 2023:37: 674–682.
  - ◦ Important paper analyzing the normative aspects of the concept of health in One Health approaches.

## Data Availability

No datasets were generated or analysed during the current study.
